# Unmasking the Silent Intruder: A Case of Spontaneous Coronary Artery Dissection

**DOI:** 10.7759/cureus.41934

**Published:** 2023-07-15

**Authors:** Chibuike C Agwuegbo, Eman N Ahmed, Serin Moideen Sheriff, Emmanuel O Olumuyide, Sahani A Waduge

**Affiliations:** 1 Internal Medicine, Western University of Health Sciences, Temecula, USA; 2 Internal Medicine, College of Medicine Alfaisal University, Riyadh, SAU; 3 Emergency Medicine, Lal Bahadur Shastri Hospital, Delhi, IND; 4 Internal Medicine, Odessa National Medical University, Odessa, UKR; 5 Internal Medicine, Insight Hospital and Medical Center, Chicago, USA; 6 Internal Medicine, University of Science and Technology, Chittagong, BGD

**Keywords:** interventional cardiology, acute coronary syndrome, coronary angiography, chest pain, coronary pathology, spontaneous coronary artery dissection

## Abstract

Spontaneous coronary artery dissection continues to pose a diagnostic dilemma in the evaluation of patients with chest pain. Our case discusses its manifestation in a male patient who visited the emergency department complaining of recent-onset chest pain. Evaluation of his chest pain through coronary angiography revealed luminal radiolucency corresponding to type 1 spontaneous coronary artery dissection (SCAD). The patient was promptly managed using medical interventions until stability was achieved and referred to cardiac rehabilitation care with close follow-up. In our literary contribution, we present a fascinating diagnosis, potentially life-threatening, observed in an otherwise active and healthy male patient. Notably, this diagnosis is uncommon in the male population. Through this study, we aimed to shed light on understanding, awareness, and clinical recognition of SCAD, ultimately improving patient care and outcomes.

## Introduction

Spontaneous coronary artery dissection (SCAD) is a non-iatrogenic, non-traumatic separation of the coronary artery wall by intramural hemorrhage. It is a rare but important and potentially fatal cause of acute coronary syndrome (ACS) [1]. SCAD may be atherosclerotic or non-atherosclerotic in origin. It has been documented in 0.1-4% of total cases of ACS, with the majority being young women with no significant risk factors or past medical history [2]. Male patients make up only 10% of all SCAD cases [3]. The most commonly affected vessels are the left anterior descending (LAD) vessels followed by the right coronary artery (RCA), and the left main coronary artery (LMCA) [4]. Here, we outline a rare presentation of SCAD in a young male with no traditional risk factors.

## Case presentation

A 36-year-old male with a known history of asthma, presented to the emergency department (ED) with complaints of chest pain. The pain occurred after a 2-hour mountain bike ride and intensified while he was walking with his wife later that evening. The patient experienced sudden, pressure-like, left-sided chest pain radiating down the arm, with no specific aggravating or relieving factors. He denied experiencing any associated symptoms, such as sweating, dizziness, abdominal pain, nausea, or vomiting. The patient had no history of smoking, alcohol consumption, or illicit drug use. Additionally, he led an active lifestyle and is employed as a sheriff.

On physical examination, the patient was in distress, conscious, and oriented to time, place, and person. His vital signs were recorded as follows: temperature 98.2°F, blood pressure 160/76 mmHg, heart rate 78 beats per minute, respiratory rate 18 breaths per minute, BMI 24.4 kg/m², and oxygen saturation 99% on room air. His mucous membranes were moist, and palpation of the abdomen was soft and non-tender. Auscultation of the chest revealed normal heart sounds without any detectable murmurs, and the examination of the bilateral lung fields indicated clear findings.

Laboratory investigation results indicated a significantly elevated troponin level at 1,761 ng/L (normal values at our institution range from 0 to 76.2 ng/L), continuously rising at 3 hours (9,402 ng/L) and 6 hours (11,790 ng/L). The lipid profile demonstrated elevated low-density lipoprotein (LDL) (141 mg/dL) and total cholesterol levels (226 mg/dL) (Table 1). The results of other laboratory tests, including complete blood count, comprehensive metabolic panel, pro-B-type natriuretic peptide (BNP), thyroid stimulating hormone, and free T4, showed no significant abnormalities.

**Table 1:** Lipid panel of the patient upon presentation revealing elevated total cholesterol, HDL, and LDL. HDL: high-density lipoprotein; LDL: low-density lipoprotein; VLDL: very low-density lipoprotein

Component	Patient value	Standard range
Total cholesterol	226	111-199 mg/dL
Triglycerides	100	34-150 mg/dL
HDL	71	40-59 mg/dL
LDL direct	141	0-100 mg/dL
VLDL	20	2-30 mg/dL
Cholesterol/HDL ratio	3.18	0-5.0

**Figure 1:**
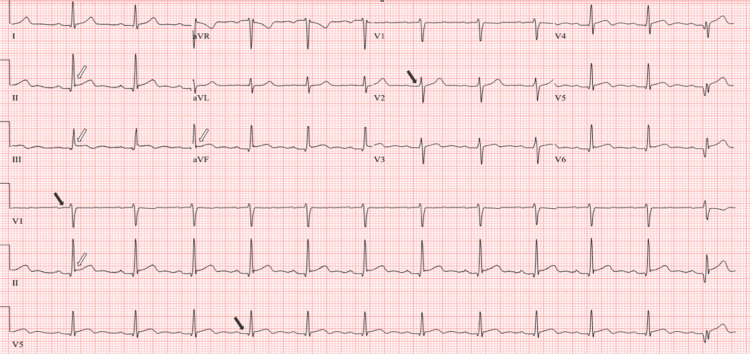
Electrocardiogram showing mild diffuse ST-segment elevations (white arrows) and first-degree atrioventricular block (black arrows).

Initial electrocardiogram (EKG) showed mild diffuse ST-segment elevations and first-degree atrioventricular block, and a repeat EKG done 5 hours later remained unchanged (Figure 1). An echocardiogram reported normal global left ventricular systolic function and size, an ejection fraction of 50-55%, normal right ventricle size and function, both left and right atria were normal in size, and all the valves were normal.

**Figure 2:**
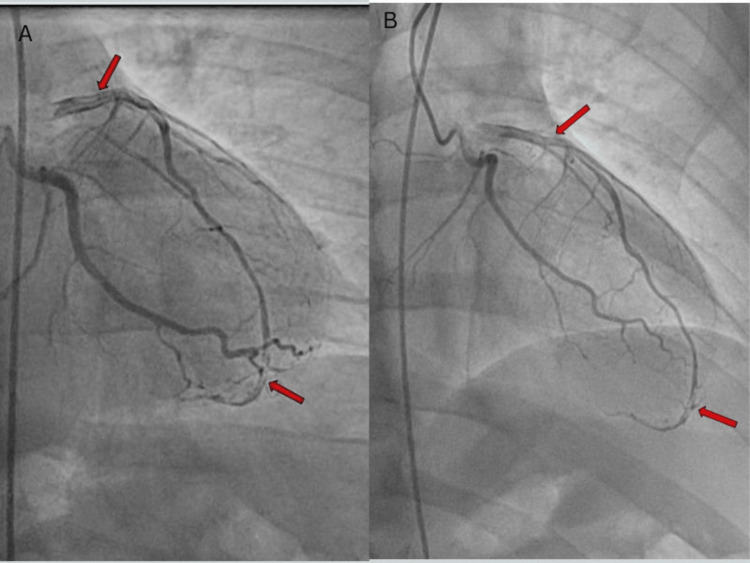
Type 1 spontaneous coronary artery dissection of the left anterior descending artery (LAD) from the proximal segment and involving the entire LAD, TIMI flow 3 (A and B). Arrows point towards multiple radiolucent lumen. TIMI: thrombolysis in myocardial infarction

Subsequently, the patient underwent a diagnostic catheterization, which revealed a left dominant circulation. It was discovered that the patient had a type 1 spontaneous coronary artery dissection in the left anterior descending artery, starting at the proximal section and extending throughout the entire artery, with thrombolysis in myocardial infarction (TIMI) flow 3 (Figure 2). All other blood vessels were found to be completely patent. Stenting was deemed too risky due to the potential for exacerbating the dissection, leading to the decision to manage the patient conservatively. The patient was medically treated with aspirin, clopidogrel, atorvastatin, and metoprolol tartrate. Effective double-product control was achieved, and the patient was advised to limit physical activity and avoid heavy lifting. Upon discharge, the patient's condition was stable, with notable improvement in symptoms. Additionally, the patient was referred to a cardiac rehabilitation program and scheduled for close outpatient follow-up.

## Discussion

Spontaneous coronary artery dissection (SCAD) is a rare disease, first diagnosed in 1931. It was often undiagnosed and discovered only during postmortem examinations. SCAD can occur in individuals without identifiable risk factors, it is commonly noted in young women between the ages of 41 and 52 years, accounting for almost a quarter of cases of ACS in women <50 years of age [5,6]. The incidence of SCAD is found in as less as 10% of reported cases in males [3]. However, SCAD should often be kept in mind when assessing a male patient with chest pain without a history of CAD, cardiac surgery, or chest trauma. Recent studies have shown that SCAD can account for 1-4% of acute coronary syndrome cases [6]. SCAD can be classified as primary or secondary, with primary SCAD having unknown causes and secondary SCAD being attributed to factors such as coronary catheterization, cardiac surgery, or chest trauma [6]. The exact pathogenesis of SCAD is not fully understood, but it involves the dissection of the coronary intima or media with hematoma formation within the vessel wall [6]. The most affected artery in SCAD is the left anterior descending artery, particularly in its mid to distal segments [4]. Some conditions have been noted to increase the risk of developing SCAD, such as connective tissue diseases, fibromuscular dysplasia, vasculitis, hormonal therapy, and post-partum period. Extreme physical exertion, emotional stress, and Valsalva straining are the usual precipitating factors [7].

Patients with sudden coronary artery dissection can present with a wide range of symptoms, including chest pain, shortness of breath, palpitations, dizziness, and sweating [7]. A high index of suspicion is crucial in making a timely and accurate diagnosis of SCAD. Diagnosis should include history, physical examination, laboratory, and imaging. The workup for SCAD starts with cardiac biomarkers, most importantly troponin, which is typically elevated, and ECG findings in SCAD patients are mostly consistent with ST-elevation myocardial infarction (STEMI) or non-ST-elevation myocardial infarction (NSTEMI) [8]. The diagnosis of SCAD is further confirmed with coronary imaging. 

Coronary angiography is the gold standard for the diagnosis of SCAD. Traditionally, the findings of SCAD on coronary angiography were defined by the presence of multiple radiolucent lumens [9]. Other coronary angiography findings have since been described; hence, an angiographic classification has been developed. Type 1 SCAD features multiple radiolucent lumens. Type 2 SCAD, which is the most common type of SCAD, refers to the presence of diffuse stenosis. Type 3 SCAD frequently mimics atherosclerosis, with the presence of focal or tubular stenosis [10]. Our patient presented with features consistent with type 1 SCAD. When diagnostic doubts persist, other intracoronary imaging modalities can be used to further diagnose SCAD. These include intravascular ultrasound, optical coherence tomography, and cardiac computed tomography angiography.

SCAD is managed with either medical or revascularization as the major modalities. While percutaneous coronary intervention (PCI) and coronary artery bypass grafting (CABG) are reserved for severe, multiple, or proximal stenosis and unstable patients, medical modalities are used in cases of hemodynamic stability and distal or mild coronary stenosis [11]. The empiric medications given are aspirin, P2Y12 inhibitors (e.g., clopidogrel, ticagrelor, and prasugrel), statins, and beta-blockers. The results of ECG, echocardiogram, a positive angiographic confirmation with low-risk TIMI flow, and risk of potential dissection outweighed dynamic troponin and active pain. Hence, our patient was directed to a conservative strategy and appropriately managed with aspirin, clopidogrel, atorvastatin, and metoprolol tartrate. There have been reports of readmission due to chest pain within 30 days of hospitalization after myocardial infarction due to SCAD in 24% of the study’s subjects [12]. The use of angiotensin-converting enzyme inhibitors (ACEIs)/angiotensin receptor blockers (ARBs) and antianginal medications such as calcium channel blockers, nitrates, or ranolazine would theoretically prove to be beneficial to prevent recurrence; however, these effects have to be studied yet. The use of ACEIs and ARBs has only been found in cases of left ventricular dysfunction [13]. Medical therapy has an excellent prognosis [14] and mortality was reported in around 1% of patients observed over 2.5 years [15]. Aside from inpatient management, these patients are advised to avoid high-intensity, strenuous exercise as these are potential physical triggers [16]. Furthermore, most of these patients are referred to cardiac rehabilitation programs to prevent recurrence or worsening of the condition [7]. With close monitoring post-management, patients’ symptoms resolve over time with arterial healing, and they are able to carry out regular activities.

## Conclusions

This study encourages the choice and successful prognosis of medically managing a young patient found to have type 1 SCAD affecting the LAD in the setting of troponin progression and active chest pain. SCAD is a rare but important and potentially life-threatening cause of ACS. Although SCAD has been traditionally described in the female population, it can also occur in male patients, as highlighted in our article. SCAD is classified based on the angiographic findings of the vessel affected and the segment of the vessel affected. Diagnosis of SCAD hinges on history, laboratory tests, and imaging, with coronary angiography being the gold standard of diagnosis. Management of SCAD could be either conservative with medical therapy, or interventional with PCI or CABG, depending on the hemodynamic status and the vessel affected. A high index of suspicion should be maintained for early identification and management of SCAD.
